# Clinical presentation and management of chromoblastomycosis: A case report and review

**DOI:** 10.1016/j.mmcr.2023.05.004

**Published:** 2023-05-27

**Authors:** Lacey Falgout, Deborah Hilton

**Affiliations:** Louisiana State University Health Sciences Center, Department of Dermatology, New Orleans, LA, USA

## 1 Introduction

Chromoblastomycosis (chromomycosis) and phaeohyphomycosis are rare, chronic granulomatous cutaneous infections of the skin and subcutaneous tissue caused by traumatic inoculation of ubiquitous dematiaceous (melanized) fungi most commonly affecting agricultural workers in tropical and subtropical climates [1]. Chromoblastomycosis was first described in the United States by Lane and Medlar in 1915. The lesions are characteristically polymorphous and may present as a small papule evolving into hyperkeratotic plaques, verrucous nodules, or deep fungating ulcerations, making it an elusive clinical diagnosis requiring a high clinical suspicion. Diagnosis is made by visualization of muriform bodies or pigmented hyphae on biopsy [2,3]. Treatment options include pharmaceutical, surgical, and chemotherapy. Patients are commonly treated with a combination treatment due to the recurrence rate of deep fungal infections. Patients who have a history of immunodeficiency or organ transplant are at a higher risk of developing opportunistic dematiaceous deep fungal infections [4].

## 2 Case

**Fig. 1:**
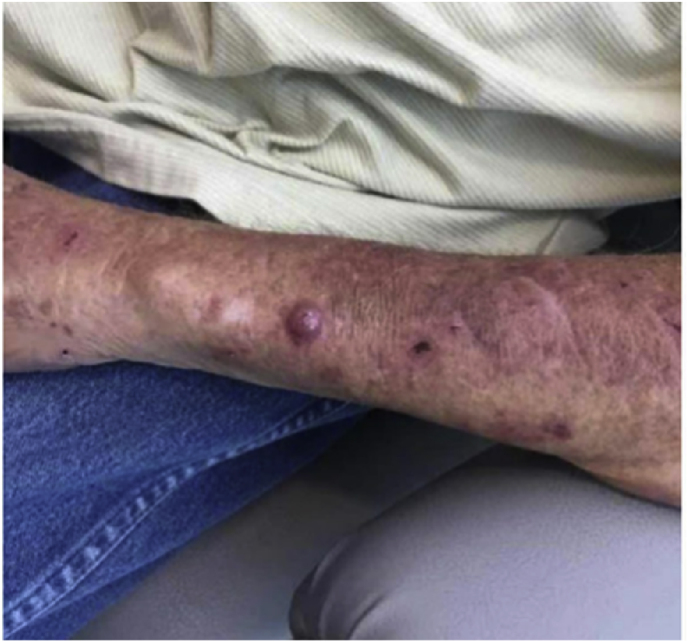
Erythematous dome-shaped papule of the left dorsal forearm, measuring 1.8 cm in diameter.

**Fig. 2:**
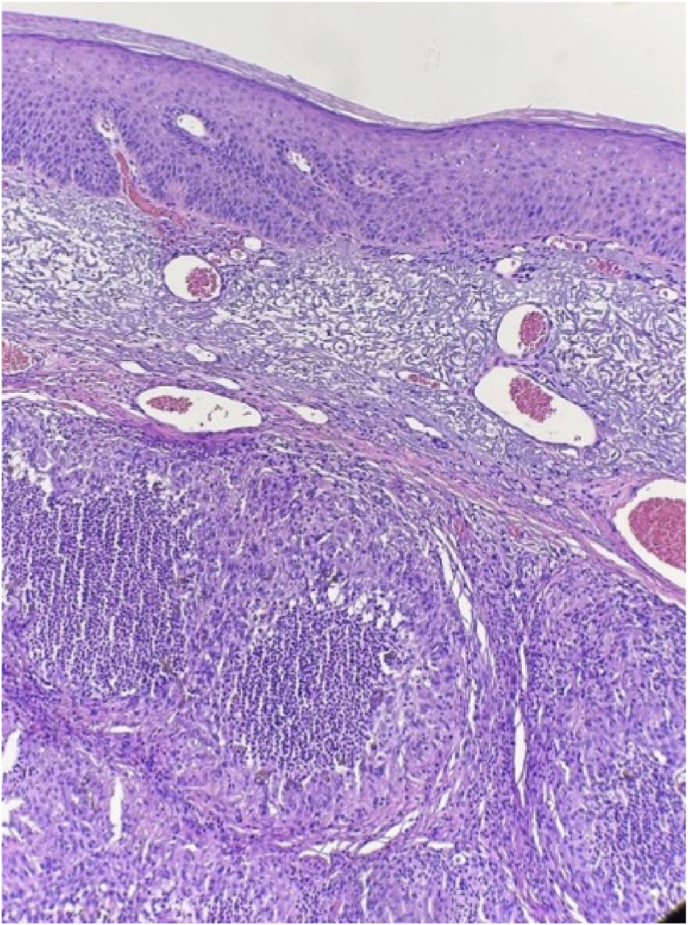
Pathology specimen from shave biopsy revealing epidermal acantholysis overlying a large nodule comprised of a granulomatous infiltrate surrounding multiple cysts.

**Fig. 3:**
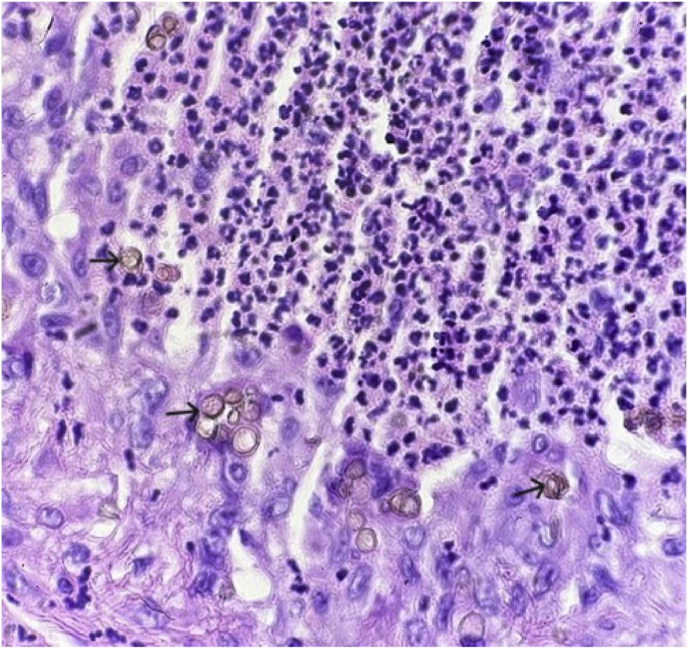
Pathology specimen from shave biopsy revealing round, thick-walled bodies within the cystic microabscesses.

We present the case of a 68-year-old male who presented with an enlarging, erythematous, non-healing lesion of concern on the left dorsal forearm that has been present for several months. Past medical history is significant for chronic kidney disease, congestive heart failure, chronic obstructive lung disease, and non-melanoma skin cancer. Physical examination revealed a tender, brightly erythematous dome-shaped papule, measuring 1.8 cm in diameter (Fig. 1). Differential diagnosis included keratoacanthoma, squamous cell carcinoma, basal cell carcinoma, and metastatic carcinoma. The patient could not recall trauma to the area. A shave biopsy was performed, with histopathology revealing epidermal acantholysis overlying a large nodule comprised of a granulomatous infiltrate surrounding multiple cysts (Fig. 2). Round, thick-walled bodies are noted within the cystic microabscesses (Fig. 3). A PAS stain highlighted the pigmented fungal cells with appropriately reactive controls, suggestive of a deep fungal infection. Fungal culture was not performed. Further history obtained from the patient revealed a hobby of gardening and raising numerous farm animals. The lesion was surgically excised with clear margins. The patient declined systemic antifungal treatment. The patient follows up every 6 months with no evidence of recurrence at 18 months following excision.

## 3 Discussion

Chromoblastomycosis and phaeohyphomycosis are cutaneous fungal infections caused by transcutaneous trauma of opportunistic dematiaceous fungi. There are over 100 species of pigmented fungi belonging to the order *Chaetothyriales* that are implicated in causing these infections. The most commonly isolated agents are *Fonsecaea pedrosoi* (90%) and *Cladophialophora carrionii*. Pigmented fungi are also denominated “dematiaceous” or “melanized” fungi because these species produce microscopically visible melanin in their cell walls. Deposition of fungus subcutaneously elicits a cascade of granulomatous inflammation involving macrophages and Langerhans cells. D'Avila et al. described a predominance of IL-4 and IL-10 in verrucoid lesions, representing a Th2 response. They also observed a predominance of IFN-gamma and TNF-alpha with well-formed granulomas in atrophic lesions, representing a Th1 response [5]. Although systemic involvement is rare, Qiu et al. describe the case of a 42-year-old immunocompetent male agriculturalist from China with skeletal and pulmonary involvement treated with systemic antifungal therapy for 20 months with improvement. This patient was ultimately lost to follow-up [6].

These infections are more prevalent in persons living in poverty-stricken tropical and subtropical zones. There is a strong association with agricultural laborers who come into contact with colonized wood or soil, though most patients do not recall an inciting trauma. The lower legs are most commonly affected. While men are most commonly affected, Canela et al. describe a 91-year-old immunocompetent female immigrant from Taiwan diagnosed in the United States with chromoblastomycosis. The patient was treated with daily itraconazole and had improvement in the size and severity of the lesion [7]. Chromoblastomycosis and phaeohyphomycosis have been identified in all continents with the exception of Antarctica, with most cases being reported from South America, Central America, and Africa.

Diagnosis is made by visualization of muriform bodies or pigmented hyphae on histopathology and/or mycological examination. Phaeohyphomycosis is indistinguishable from chromoblastomycosis clinically, both of which are deep cutaneous infections caused by the same types of pigmented fungi. The difference between the two can be appreciated histologically. The characteristic histopathology of phaeohyphomycosis demonstrates clear melanized hyphal forms in the epidermis and dermis. Chromoblastomycosis instead demonstrates muriform bodies within multinucleated giant cells in the subcutaneous tissue and may demonstrate hyphal forms in the stratum corneum in cases of hyperkeratosis. Fungal culture may be utilized to isolate and identify causative species.

Clinically the lesions are polymorphous, presenting as a papule that may evolve into hyperkeratotic plaques, verrucous nodules, or fungating ulcerations. This represents a diagnostic challenge that requires a high clinical suspicion for diagnosis. Treatment choice may depend on the location and extent of the lesions with options including pharmaceutical, surgical, chemotherapy, immunotherapy, and cryotherapy. Surgical excision may be most helpful in cases of a single, primary lesion with confirmation of clear margins. Patients are commonly treated with a multimodal approach due to the recalcitrant nature of implantation fungal infections.

Systemic antifungal therapy with itraconazole (200–400mg daily) or terbinafine (500–1000mg daily) for 6–12 months may be curative in mild to moderate cases. In more severe cases, systemic treatment may be helpful in reducing the size of the lesion to allow for surgical excision. Pulse itraconazole therapy (400 mg/day for 7 days/month) has been effective in the treatment of chromoblastomycosis and may offer a more economical therapy option with better compliance [8].

Second-generation triazoles such as voriconazole and posaconazole have shown in vitro antifungal activity against the etiological agents. Negroni et al. reported five of six patients with disease refractory to standard antifungal therapy achieved cure with the use of posaconazole [9]. Lima et al. reported marked improvement in disease severity of a 48-year-old male with severe and extensive chromoblastomycosis who previously failed treatment with itraconazole, terbinafine, and cryosurgery [10]. Posaconazole and voriconazole are promising for the treatment of implantation mycoses, but currently remain cost-prohibitive for many patients.

## 4 Conclusion

Diagnosis of chromoblastomycosis and phaeohyphomycosis requires a high clinical suspicion and histopathologic examination. Traditionally this type of opportunistic mycosis has been more commonly seen in agricultural workers in the tropics and subtropics, however there are increasing reports worldwide with varying fungal species and resistance patterns associated with specific regions. Chromoblastomycosis and phaeohyphomycosis are notably difficult to treat with a high recurrence rate. Early identification and treatment are imperative to decrease the risk of further complications such as dissemination, secondary bacterial infection, or carcinomatous degeneration.

## Funding

None.

## Medical Mycology Case Reports ETHICAL FORM

Medical Mycology Case Reports requires full disclosure of all sources of funding and potential conflicts of interest. The journal also requires a declaration that the author(s) have obtained written and signed consent to publish the case report from the patient or legal guardian(s).

If you have nothing to declare in any of these categories then this should be stated.

## Funding source

All sources of funding should be acknowledged and you should declare any extra funding you have received for academic research of this work. If there are none state ‘there are none’.

Please state any sources of funding for your research:

No funding

## Consent

Please declare that you have obtained written and signed consent to publish the case report from the patient or legal guardian(s).

Please state that consent has been obtained from the patient or legal guardian(s):

Written informed consent was obtained from the patient or legal guardian(s) for publication of this case report and accompanying images. A copy of the written consent is available for review by the Editor-in-Chief of this journal on request.

As corresponding author, I hereby declare that I sign this document on behalf of all the authors of the above mentioned manuscript.

## Declaration of competing interest

None.
